# New Findings for Maternal Mortality Age Patterns: Aggregated Results for 38 Countries

**DOI:** 10.1371/journal.pone.0059864

**Published:** 2013-04-16

**Authors:** Ann K. Blanc, William Winfrey, John Ross

**Affiliations:** 1 Population Council, New York, New York, United States of America; 2 Futures Institute, Glastonbury, Connecticut, United States of America; 3 Futures Group, Senior Fellow, Washington, D.C., United States of America; Tulane University School of Public Health and Tropical Medicine, United States of America

## Abstract

**Background:**

With recent results showing a global decline in overall maternal mortality during the last two decades and with the target date for achieving the Millennium Development Goals only four years away, the question of how to continue or even accelerate the decline has become more pressing. By knowing where the risk is highest as well as where the numbers of deaths are greatest, it may be possible to re-direct resources and fine-tune strategies for greater effectiveness in efforts to reduce maternal mortality.

**Methods:**

We aggregate data from 38 Demographic and Health Surveys that included a maternal mortality module and were conducted in 2000 or later to produce maternal mortality ratios, rates, and numbers of deaths by five year age groups, separately by residence, region, and overall mortality level.

**Findings:**

The age pattern of maternal mortality is broadly similar across regions, type of place of residence, and overall level of maternal mortality. A “J” shaped curve, with markedly higher risk after age 30, is evident in all groups. We find that the excess risk among adolescents is of a much lower magnitude than is generally assumed. The oldest age groups appear to be especially resistant to change. We also find evidence of extremely elevated risk among older mothers in countries with high levels of HIV prevalence.

**Conclusions:**

The largest number of deaths occurs in the age groups from 20-34, largely because those are the ages at which women are most likely to give birth so efforts directed at this group would most effectively reduce the number of deaths. Yet equity considerations suggest that efforts also be directed toward those most at risk, i.e., older women and adolescents. Because women are at risk each time they become pregnant, fulfilling the substantial unmet need for contraception is a cross-cutting strategy that can address both effectiveness and equity concerns.

## Introduction

With recent results showing a global decline in overall maternal mortality during the last two decades [1], [2] and with the target date for achieving the Millennium Development Goals only three years away, the question of how to continue or even accelerate the decline has become more pressing. By knowing where the risk is highest as well as where the numbers of deaths are greatest, it may be possible to re-direct resources and fine-tune greater effectiveness in efforts to reduce maternal mortality reduction.

An important aspect of designing programs is knowing who is most at risk, and the age pattern sharply differentiates risk. It is expected to be roughly “J” shaped for a number of interrelated biological and social reasons – many of them overlap, though not all are well understood. For example, it is difficult to separate the effects of age from those of parity. The risk of death among young mothers may be influenced by incomplete pelvic growth, leading to a greater probability of obstructed labor, while among older women existing morbidities may complicate pregnancy. Although most women experiencing life threatening obstetrical hemorrhage have no clear risk factors, it tends to be more common among women over 40 and women under 20, although not all studies find age to be a significant risk factor [3]–[6]. Pregnancy induced hypertension is also more common among older women. Younger and older women are less likely to receive antenatal care than women age 20–34 [7] and older women are less likely to deliver with a skilled attendant [8]. Another confounding factor is the correlation between having early and late births with low socioeconomic status.

In the absence of reliable civil registration data, the large majority of developing countries rely on survey data to estimate overall maternal mortality levels [2]. However, few surveys employ large enough samples to reliably estimate age-specific rates and ratios. In this paper, we aggregate data from 38 Demographic and Health Surveys (DHS) to produce age schedules of maternal mortality for developing countries as a whole, and for three regions (sub-Saharan Africa, and selected countries in Asia, and Latin America); for urban-rural differences, and for high vs. low mortality countries. For each subgroup the mortality ratios and rates are given, as well as the proportionate share of all deaths. To our knowledge, this is the first time that age patterns for numerous countries have been presented since a 1974 article by Nortman [9].

## Methods

### Calculation of maternal mortality rates and ratios for individual countries

Thirty-eight Demographic and Health Surveys that included a maternal mortality module and were conducted in 2000 or later were included in this analysis. We calculated nearly all of the maternal mortality rates using the direct sisterhood method based upon seven years of recall. Seven years avoids heaping that occurs with five or ten year recall periods. Also, seven years is the most frequently used recall period in the DHS series, thus allowing greater ability to compare our findings with published results. Pakistan is the exception; the survey instrument and data file were different from the other surveys so the necessary data were taken from the final DHS report; the recall period is three years.

The sisterhood method depends on some well-known assumptions: women can report accurately on their siblings, there is no relationship between the number of siblings and mortality risk, fertility patterns have not changed by age, and the age pattern of respondents approximates that of the siblings [10]. It is also generally agreed that the direct sisterhood method leads to a systematic under-estimate of maternal mortality [2]. In spite of these short-comings this method uses data that are readily available for many of the countries with the largest burden of maternal mortality.

For each country, we constructed two data sets: one comprises mortality data and one comprises fertility data. The observations in the mortality data set derive from the sisters of women interviewed in the woman's questionnaire of the DHS. Each woman was asked a series of questions about each sister's survival status, current age or age at death and years since death, and whether the death occurred during pregnancy, childbirth, or within two months after the end of pregnancy or childbirth [11]. From these data, we tabulated the number of deaths of sisters in a particular five year age group (except for ages above 40 where we combine two five year age groups due to small numbers) whose death occurred during pregnancy, at childbirth or within two months of a child's birth. The woman-years of exposure are the number of years a sister was alive during the seven year recall period for a specific age group. For example, a living sister who was 28 years old at the time of the survey would contribute four years of exposure to the 25–29 year age group and three years to the 20–24 year age group.

Using the direct method of calculation, the sisters' maternal mortality rate (deaths per 1000 years of exposure) is the number of maternal deaths of sisters in a given age group multiplied by 1000 divided by their women-years of exposure. Then the age-specific maternal mortality ratio (MMR) is the mortality rate of sisters divided by the age specific fertility rate among survey respondents. The age-specific fertility rates came from the second data set, constructed for a seven year recall period using the birth history collected in the woman's questionnaire of the DHS.

The assignment of urban or rural status to a sister is based upon the residence of the respondent who reported on that sister. If the sister is not living in the same area as the respondent there can be a misallocation of her residence.

As a check on the methodology we attempted to replicate the overall maternal mortality ratio as published in the DHS final reports. Therefore, in addition to calculating the maternal mortality rates and ratios for a seven year recall period, we also calculated the rates and ratios for the particular recall period reported in the final DHS report for the same country, which often differed from the seven year period we used. Out of 38 calculations, 33 show less than 5 percent difference between the DHS published value and our calculation. In four cases the difference was between 5 and 10 percent. In the case of Bangladesh the difference was 13 percent. Peru was omitted from this confirmatory exercise for lack of a published MMR from the DHS data set.

### Calculation of aggregated maternal mortality rates and ratios

To obtain aggregated rates and ratios for country subgroups, we used weights derived from two factors: the weight assigned to the woman based upon the survey sampling design, and a scaling factor proportional to the size of the country relative to women- years of exposure in the survey. The scaling factor is the following fraction, specific to each age group:
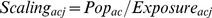
(1)Where:

*Pop_ac_* is the population of women in age group (a), in country (c), in 2005;

*Exposure_acj_* is calculated years of exposure for women in age (a) in survey (c) for module (j) to designate the woman's (i.e., respondents') survey used for the fertility rates or the maternal mortality module for which the sisters are the observational units.

The scaling factor is then used in combination with the survey sample weight to establish the weight for a particular observation. The weight assigned to a particular woman is therefore:
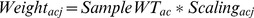
(2)


The result is that the aggregated rates and ratios are weighted according to the size of the population of women of reproductive age in each country; countries with large populations are weighted proportionately more heavily than those with small populations.

For the urban and rural estimates there is a further indexing of the equations by urban and rural to obtain suitable weightings.

The steps above make it possible to combine deaths and sister-exposure across countries to calculate aggregate age-specific maternal mortality rates; and to calculate age-specific fertility rates (ASFRs) from births and respondent exposure times. The ratios then come from dividing the rates by the ASFRs.

Table 1 presents the numbers of surveys and deaths, and the proportion of women age 15–49 in each region covered in the analysis. The majority of the surveys were conducted in sub-Saharan Africa, which is reasonably well represented with 76 percent of the population covered. The five Asian surveys cover about 40 percent of the population of Asia minus India and China. Latin America is represented by only four countries, comprising only about ten percent of the region; we use the “region” designation for convenience but recognize the limitations of the available data.

**Table 1:** Number of surveys, maternal deaths, and percent of population of women age 15–49 covered by the data.

	Number of surveys	No. of maternal deaths reported (1)	Percent of women aged 15–49 covered by survey countries	
All	38	5,247	42.2%	(2)
Sub-Saharan Africa	29	3,809	75.7%	(3)
Latin America	4	297	9.7%	
Asia ex. China and India	5	1,141	39.5%	(4)

(1) weighted by survey sample weights.

(2) Less developed regions excluding China and India.

(3) Morocco is included in the count in col. 1 as the only African member not in sub-Saharan Africa, but is omitted in the other two columns.

(4) Asia excluding China and India.

## Results

Three sets of age-specific results are provided in Table 2: maternal mortality ratios (MMRs), maternal mortality rates (MM Rates), and the distributions of numbers of deaths. Each set is provided for all countries combined, for three regional groups, for urban and rural, and for two groups: one that comprises countries where the overall MMR is >500 (“High Group”), and another that comprises countries where the overall MMR is <500 (“Low Group”). Table 3 provides the country-specific numbers of deaths, exposure times, and overall MM Rates.

**Table 2:** Maternal Mortality Ratios and Rates, and Percent Distribution of Deaths, by Age.

Maternal Age							
Maternal Mortality Ratios (deaths per 100,000 births)	15–19	20–24	25–29	30–34	35–39	40–49	All
All	408	319	411	525	740	1351	477
Sub Saharan Africa	504	416	508	672	842	1411	589
Asia	269	201	302	334	599	1240	332
Latin America	190	164	203	360	549	1167	292
Low MMR Group	288	218	316	366	654	1249	357
High MMR Group	561	466	550	741	847	1445	641
Urban	379	300	383	388	621	*	341
Rural	429	347	461	641	778	1353	595

* Insufficient sample size							
Maternal Mortality Rates (deaths per year per 1000 women age 15–49)	15–19	20–24	25–29	30–34	35–39	40–49	All
All	0.43	0.65	0.85	0.90	0.82	0.49	0.66
Sub Saharan Africa	0.68	1.05	1.32	1.57	1.40	0.91	1.09
Asia	0.22	0.34	0.51	0.43	0.45	0.24	0.35
Latin America	0.15	0.25	0.30	0.42	0.44	0.25	0.28
Low MMR Group	0.27	0.40	0.57	0.52	0.57	0.30	0.42
High MMR Group	0.74	1.16	1.44	1.77	1.45	0.97	1.19
Urban	0.30	0.47	0.65	0.55	0.52	0.41	0.47
Rural	0.57	0.82	1.01	1.18	0.98	0.60	0.83

Percent Distribution of Maternal Deaths	15–19	20–24	25–29	30–34	35–39	40–49	All
All	14.2%	18.9%	21.0%	18.6%	14.2%	13.1%	100.0%
Sub Saharan Africa	15.0%	19.5%	20.1%	19.1%	13.5%	12.9%	100.0%
Asia	12.4%	17.7%	23.5%	17.4%	15.6%	13.3%	100.0%
Latin America	10.7%	16.0%	16.9%	20.6%	19.0%	16.9%	100.0%
Low MMR Group	13.1%	17.7%	22.2%	17.4%	16.1%	13.5%	100.0%
High MMR Group	15.0%	19.8%	20.1%	19.6%	12.8%	12.8%	100.0%
Urban	13.1%	19.8%	23.2%	16.4%	12.8%	14.7%	100.0%
Rural	15.2%	18.8%	20.0%	19.5%	13.6%	13.0%	100.0%

**Table 3:** Numbers of Deaths, Exposure Times, and Maternal Mortality Rates, by High and Low MMR Levels.

	Survey Date	Region	MMR Level*	Survey deaths**	Exposure Times***	MM Rate****
Cameroon	2004	Africa	HIGH	153	124,309	1.23
Chad	2004	Africa	HIGH	176	67,946	2.59
Congo	2005	Africa	HIGH	114	90,381	1.27
D.R. Congo	2007	Africa	HIGH	127	113,947	1.11
Ethiopia	2005	Africa	HIGH	197	147,001	1.34
Gabon	2000	Africa	HIGH	60	73,953	0.81
Guinea	2005	Africa	HIGH	157	77,003	2.04
Haiti	2005–2006	LAC	HIGH	103	119,864	0.86
Lesotho	2004	Africa	HIGH	82	70,058	1.17
Liberia	2007	Africa	HIGH	127	73,761	1.72
Malawi	2004	Africa	HIGH	240	121,147	1.98
Niger	2006	Africa	HIGH	178	102,892	1.73
Nigeria	2008	Africa	HIGH	398	375,746	1.06
Rwanda	2005	Africa	HIGH	178	130,757	1.36
Sierra Leone	2008	Africa	HIGH	97	61,653	1.57
Swaziland	2006–2007	Africa	HIGH	48	55,334	0.86
Tanzania	2004–2005	Africa	HIGH	156	134,269	1.16
Zambia	2007	Africa	HIGH	106	86,485	1.22
Zimbabwe	2005–2006	Africa	HIGH	98	111,080	0.89
Bangladesh	2001	Asia	LOW	688	1,140,617	0.60
Benin	2006	Africa	LOW	159	206,122	0.77
Bolivia	2008	LAC	LOW	56	183,931	0.30
Cambodia	2005	Asia	LOW	98	189,161	0.52
Dominican Republic	2007	LAC	LOW	54	349,229	0.15
Ghana	2007	Africa	LOW	79	118,953	0.66
Indonesia	2007	Asia	LOW	65	374,775	0.17
Kenya	2008–2009	Africa	LOW	99	116,632	0.85
Madagascar	2008–2009	Africa	LOW	190	230,109	0.83
Mali	2006	Africa	LOW	168	154,389	1.09
Morocco	2003–2004	Africa	LOW	58	248,312	0.24
Mozambique	2003	Africa	LOW	121	131,606	0.92
Namibia	2006–2007	Africa	LOW	70	119,793	0.58
Nepal	2006	Asia	LOW	39	111,253	0.35
Pakistan	2006–2007	Asia	LOW	192	481,127	0.40
Peru	2004–2008	LAC	LOW	84	497,816	0.17
Sao Tome and Principe	2008–2009	Africa	LOW	6	38,005	0.17
Senegal	2005	Africa	LOW	121	175,588	0.69
Uganda	2006	Africa	LOW	105	106,848	0.98
Totals			High	2,795	2,137,586	1.23
			Low	2,452	4,974,266	0.49
			All	5,247	7,111,852	0.73

Notes.

* Above or below MMR of 500.

** No. of deaths in DHS surveys, as weighted in the survey.

*** Age-specific exposure times for sisters of respondents.

**** Deaths per 1000 women age 15–44 per year.

### Maternal Mortality Ratios (MMRs)

**Figure 1:**
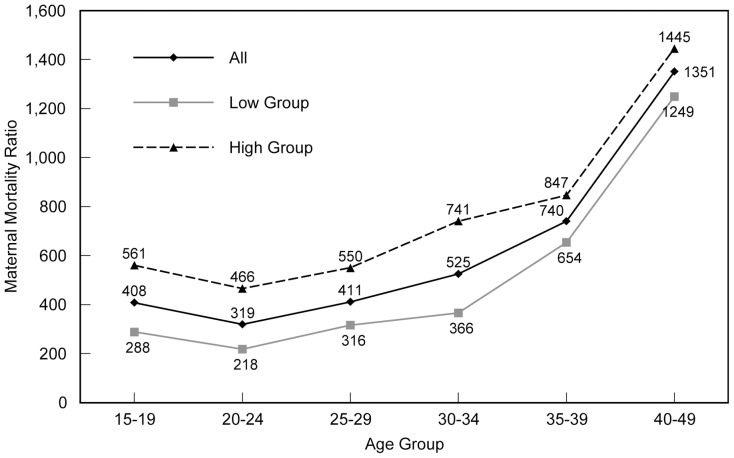
Maternal mortality ratios, all countries and low and high MMR groups. The maternal mortality ratio (MMR) is the number of maternal deaths per 100,000 births. The maternal mortality ratio by age is shown for 38 developing countries combined (All), and for two sub-groups: countries where the overall MMR is >500 (High Group), and another that comprises countries where the overall MMR is <500 (Low Group).

The maternal mortality ratio starts low and rises steeply and non-linearly after age 30 (Figure 1); the MMR curve becomes progressively steeper as age advances. The ratio starts just above 400, dips to 319 among women age 20–24, and then rises to 1351 in the oldest age group. Contrary to expectations, the age curve shows only a modest excess risk at age 15–19 compared to age 20–24. The risk for women age 15–19 is approximately 28 percent higher than for women age 20–24. The higher risk for adolescent women is relatively greater in the low MMR group than in the high MMR group.

Comparing countries with high vs. low levels of mortality, the patterns for the two groups change systematically. With the exception of the 30–34 age group, there is a regular shift in the size of the gap in moving from the youngest to oldest ages. The gap between the two groups at age 15–19 is 273 points, declining to 248 and 234 in the next two age groups, then (skipping the 30–34 group) to 193 and 196 for the two oldest groups. This result suggests a different dynamic of risk among older women.

It also suggests that as the MMR declines, the older age groups decline most slowly, since the difference between the high and the low group is least above age 35. That is reinforced when the countries are divided into quartiles by the overall MMR. The smallest differences between the top and bottom quartiles occur at ages 35–39 and 40–49: only a 58% difference vs. a 70% difference at ages 20–24 and 25–29, suggesting that the barriers to improved maternal health outcomes at the upper ages are more resistant to change.

By region, women in the sub-Saharan African countries experience much higher mortality risks at every age than those in the Asian or Latin American countries that are included (Table 2). The gap is about the same at every age except at 40–49, where there is again a suggestion of a smaller difference. Otherwise, the three regional patterns are similar; only the levels differ, indicating that there is a similarity in the causes of maternal mortality across countries as the risks increase with age, although better representation of Asian and Latin American countries would be needed to confirm this conclusion.

**Figure 2:**
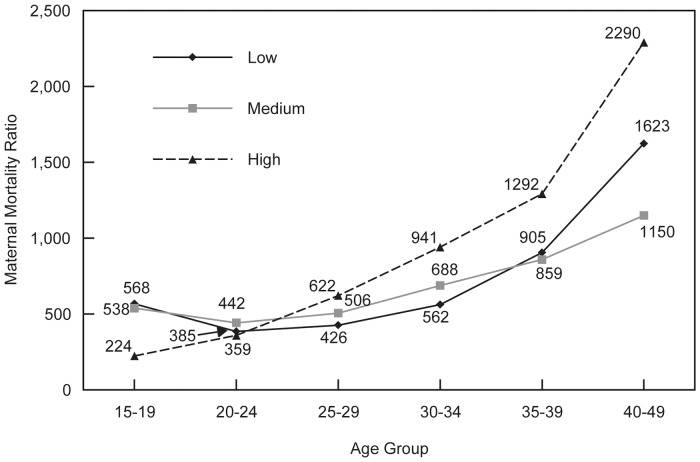
Maternal mortality ratios, sub-Saharan African countries by HIV prevalence. The maternal mortality ratio (MMR) is the number of maternal deaths per 100,000 births. Sub-Saharan African countries are grouped by the overall level of HIV prevalence in 2006 (the median year of the surveys analyzed). Countries are grouped into: high (>10%), medium (2–10%), and low (<2%) prevalence.

Within sub-Saharan Africa, we grouped countries by the overall level of HIV prevalence in 2006 (the median year of the surveys analyzed). Countries are grouped into: high (>10%), medium (2–10%), and low (<2%) prevalence. The age pattern of maternal mortality differs substantially in the group of countries with high HIV prevalence (Malawi, Mozambique, Lesotho, Namibia, Swaziland, Zambia, Zimbabwe) compared to the pattern in the other two groups (Figure 2). The MMR in the age group 15–19 is lower but then increases above age 25 and is substantially higher than the other two groups above age 30, with particularly high levels above age 40. Since the maternal deaths in the direct sisterhood method are defined in terms of time relative to a birth rather than to a specific cause, a proportion of female deaths during this window of time in countries with high prevalence are due to HIV/AIDS, either directly or indirectly. The association between HIV and maternal mortality is not well understood, but there is some evidence that HIV can increase the likelihood of maternal complications, such as hemorrhage and sepsis, and there have been a number of attempts to quantify the contribution of HIV to the number of maternal deaths globally [12]. Figure 2 suggests that the elevated risk of maternal death in countries with high HIV prevalence may occur primarily among older mothers who already are subject to high risk relative to younger mothers. Confirmation of this age pattern and its underlying causes deserves further research attention.

As expected, rural MMRs are higher than urban ones, but the difference is not great until age 30–34, where the urban point is lower than the pattern suggests, rising again at age 35–39. The anomaly may be partly due to the small numbers of women exposed to mortality risk in urban areas, and especially so at age 40–49.We are unable to confirm from these data that the underlying age patterns are similar in urban and rural areas, though that seems likely.

### Maternal Mortality Rates

The MM Rate (maternal deaths per year per 1000 women aged 15–49) is high where the risk per birth is high and/or where there are many births per woman. The rate is relatively low at the highest ages even though the risk is high, because few births occur at those ages. It is low at ages 15-19 because there are few births and the risk is low. The rate rises through age 30–34, primarily because the numbers of births are greatest there. The rates vary from 0.28 to 1.09 across the regions and average 0.66 overall, considerably less than one death per woman per year.

**Figure 3:**
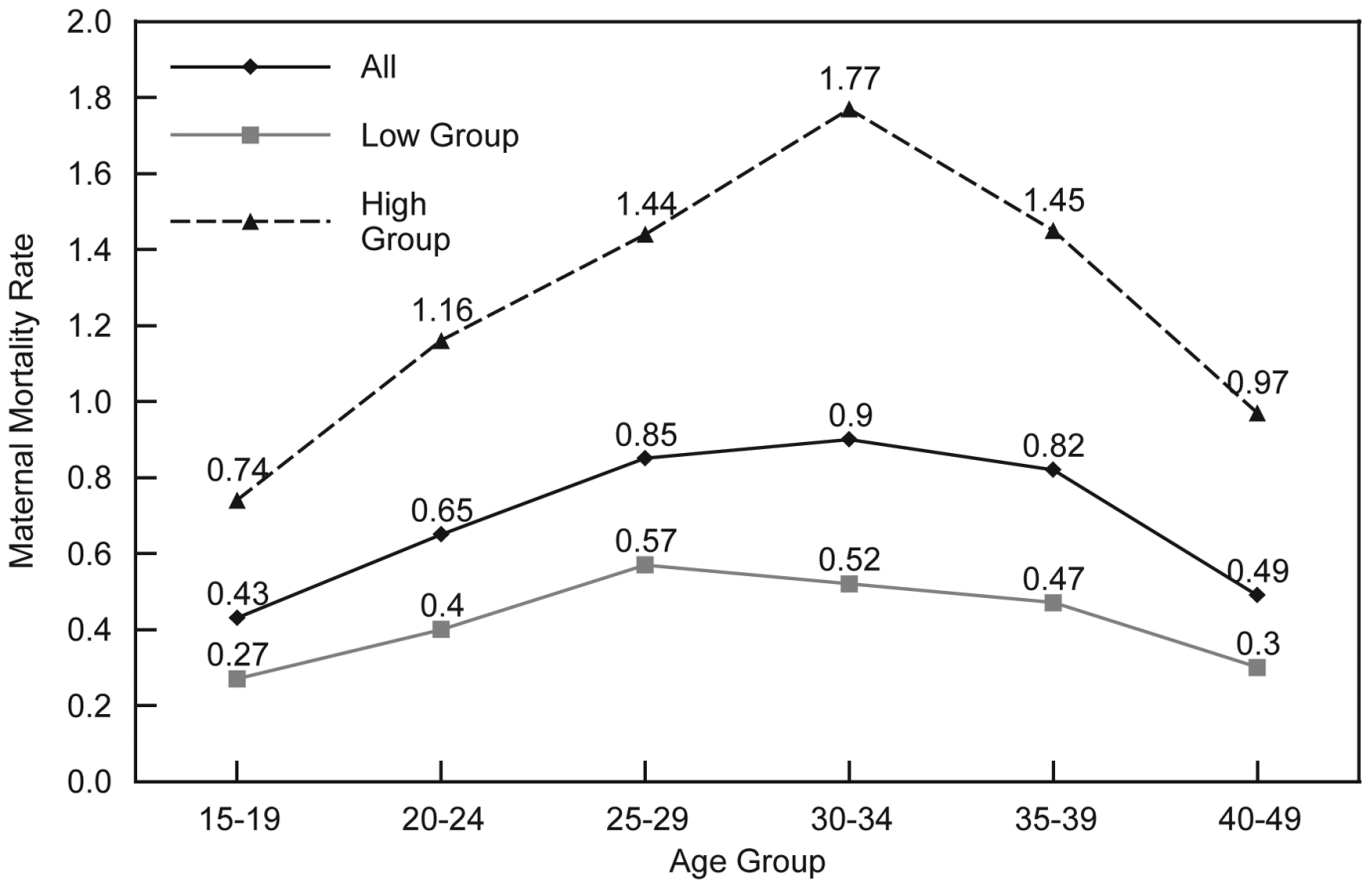
Maternal mortality rates, all countries and low and high MMR groups. The maternal mortality rate is the number of maternal deaths per 1,000 women of reproductive age (15–49). The maternal mortality rate by age is shown for 38 developing countries combined (All), and for two sub-groups: countries where the overall maternal mortality ratio (MMR) is >500 (High Group), and another that comprises countries where the overall MMR is <500 (Low Group).

The reversed U-shape pattern for rates by age appears in Figure 3, which again displays the contrast between the high and low groups by their overall MMR levels. The gap between them increases steadily to the middle of the age range and then diminishes. (It would do so even without the slightly depressed point at 30–34 for the low group.) The gap at age 30–34 results from two factors: the largest difference in both the ASFRs and in the MMRs occurs in that age group.

The rates are far higher in sub-Saharan Africa than elsewhere (Table 2). The other two regional groups, each with a small number of countries, are not much different in their averages. The pattern for the four Latin American countries however, differs from that for the five Asian countries.

By residence, the mortality rate is as expected higher at every age in the rural sector; it is approximately double the urban rate at ages 15–19 and at 30–39. When charted, the two curves are similar, except that the urban line is depressed at age 30–34.

### Numbers of Maternal Deaths

Deaths are distributed by age in a manner that parallels the patterns for MM Rates since more deaths occur where there are either more births or where the risk per birth is higher. However, another factor enters in, namely the number of women of reproductive age, which declines regularly by age.

**Figure 4:**
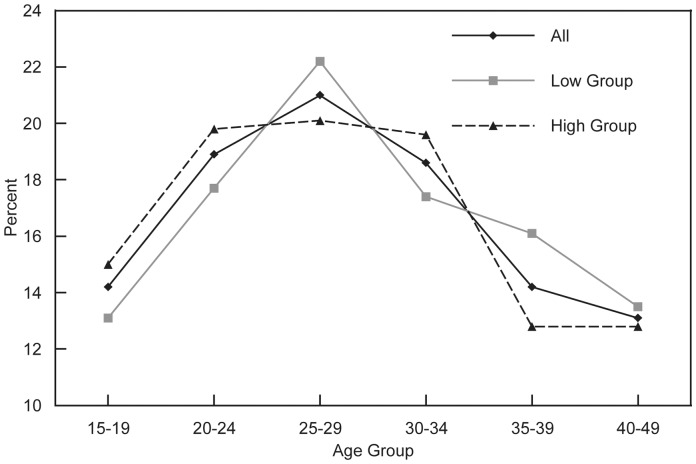
Percent distribution of maternal deaths for all countries, and low and high MMR groups. The percent distribution is the percentage of total maternal deaths among women age 15–49 that occur in each five year age group. The distribution by age is shown for 38 developing countries combined (All), and for two sub-groups: countries where the overall maternal mortality ratio (MMR) is >500 (High Group), and another that comprises countries where the overall MMR is <500 (Low Group).

The combination of these factors produces the proportionate distributions in Figure 4. Apart from the issue of total deaths, which of course tend to be lower where MMRs are lower, in both the Low and High Groups the deaths are highly concentrated in the intermediate ages, 20–24 to 30–34. The difference is that in the Low Group they peak somewhat more sharply at ages 25–29 (22% vs. 20%), and the deaths in the High Group are spread relatively evenly among ages 20–34, with a sharp decline at 35–39. The lowest percentages are at the youngest and the oldest ages.

Deaths in the Asian and sub-Saharan African countries are concentrated in the intermediate ages, between 20–24 and 30–34, and they account for most of the 38 countries in the study. The Latin American countries show a distribution that is more concentrated at age 30–34 and above.

Somewhat surprisingly, the distributions of deaths between urban and rural areas are similar, except that more urban deaths occur at age 25–29 and fewer at age 30–34. All the above figures are influenced variously by the level of risk, the fertility rate, and the relative numbers of women, depending upon the sub-groups involved.

## Discussion

By aggregating data from 38 surveys, we have shown that the age pattern of maternal mortality is broadly similar across regions, type of place of residence, and overall level of mortality. A “J” shaped curve, with markedly higher risk after age 30, is evident in all groups. That suggests that a common set of explanatory factors – both biological and social – operates across regions and other groupings. The MMRs rise dramatically at the upper ages partly because the women who get pregnant when older are selected for a number of factors related to higher mortality, including poverty and low education levels, both of which are correlated with greater numbers of children. A number of cross-country descriptive analyses have shown that women over age 35 or 40 are less likely to attend antenatal care [13], have skilled attendance at birth [8], and postpartum care [14] compared to women in their twenties and early thirties. Coverage of antenatal care and skilled attendance at births declines with increasing age of the mother in virtually all of the countries included here (data not shown). It is likely that these women's access to other types of services such as emergency obstetric care and HIV/AIDS treatment are lower as well. All this suggests that the barriers to improved maternal health outcomes at the upper ages are more resistant to change. In countries with high HIV prevalence, there is evidence of very high risk of maternal mortality above age 30. Moreover there is considerable suggestion in the data that the older age groups benefit least as overall mortality declines.

The literature on maternal mortality, as well as advocacy efforts, has often stressed excessive risk among adolescent women. The most common assertion is that girls age 15–19 are twice as likely to die from childbirth as women in their twenties, while those under 15 are five times as likely to die [15]–[19]. However, our extensive review of the literature found very limited empirical support for excessive risk of this magnitude among women age 15–19. In one compilation of 13 data sets, the MMR ratio between age 15–19 and 20–24 was actually equal or below 1.0 in six sets, below 1.26 in six, at 1.5 in one [20]. Overall, in these data, the risk of death per birth for women age 15–19 is just 28 percent higher than among women age 20–24. The greatest number of maternal deaths occurs at age 25–29. The highest MM Rate comes at age 30–34. The highest risk per birth, the MMR, occurs at ages above 40. These findings indicate that special emphases heretofore on adolescent maternal mortality in advocacy and programming have been somewhat overstated. Internationally, the campaign to reduce the absolute numbers of deaths must focus heavily upon the 25–29 group and the two adjoining age groups.

There are a number of limitations in this work. First, the relative lack of data from countries in Asia (notably India and China) and Latin America means that the estimates are skewed toward sub-Saharan Africa. Second, in spite of the aggregation of multiple surveys, the few urban deaths reported above age 40 limits accurate age breakdowns in the residential analysis. We expect that improved estimates will be possible as more data become available. However, the unavoidable assignment of place of residence based on the respondent's location rather than that of her sister(s) introduces an unknown level of error in the data. Third, there are some unexplained anomalies in the age pattern, and certain curves are not as smooth as would be expected. These may be due to deficiencies in the quality of the data, which rely on past events reported by proxies who may not know the full details. We attempted to use the DHS data to estimate parity-specific maternal mortality but obtained implausible results among women at parities 0 and 1 which we believe may reflect respondent confusion about the wording of the survey question on parity.

The implications of these results for maternal health programming are not straightforward. The largest number of deaths occurs in the age groups 20–34, largely because those are the ages at which women are most likely to give birth, so efforts directed at this group would most effectively reduce the number of deaths. A similar argument can be made for concentrating resources in those ten or so countries in which the vast majority of maternal deaths occur. Yet, equity considerations indicate that maternal health efforts be directed to those older women because they are most at-risk; and to adolescents because improved adolescent health outcomes have long-term and wide-reaching development benefits.

A recently published analysis attributes roughly half of the decline in maternal deaths over the last two decades to declines in fertility levels in the developing world [21]. What is clear is that women are at risk each time they become pregnant so fulfilling the substantial unmet need for a broad array of contraception options is a cross-cutting strategy that can address both effectiveness and equity concerns.
